# Efficacy and safety of low-dose corticosteroids combined with leflunomide for progressive IgA nephropathy: a systematic review and meta-analysis

**DOI:** 10.1186/s12894-024-01438-3

**Published:** 2024-03-11

**Authors:** Dongxu Zhang, Bowen Xia, Xin Zhang, Pu Liang, Xiaopeng Hu

**Affiliations:** 1 Department of Urology, Beijing Chaoyang Hospital, Capital Medical University, Beijing, China; 2 Institute of Urology, Capital Medical University, Beijing, China; 3 Beijing Key Laboratory of Emerging Infectious Diseases, Institute of Infectious Diseases, Beijing Ditan Hospital, Capital Medical University, Beijing, China; 4 Beijing Institute of Infectious Diseases, Beijing, China; 5 National Center for Infectious Diseases, Beijing Ditan Hospital, Capital Medical University, Beijing, 100015 P.R. China

**Keywords:** Meta-analysis, IgA nephropathy, Leflunomide, Corticosteroids, Proteinuria

## Abstract

**Background and objective:**

The effectiveness of immunosuppressive and corticosteroid treatments for Immunoglobulin A (IgA) nephropathy (IgAN) remains thoroughly evaluated. We undertook a meta-analysis to investigate the efficacy and safety of low-dose corticosteroids plus leflunomide for progressive IgA nephropathy.

**Methods:**

Eligible studies were obtained from PubMed, Embase, and Cochrane Library databases. We also searched the references of the included studies. Our protocol followed the preferred reporting items for systematic reviews and meta-analyses (PRISMA) checklist. Eligibility criteria were defined using a PICOS framework.

**Results:**

Our study included three articles presenting 342 patient cases. Findings revealed that low-dose corticosteroids combined with the leflunomide group were effective in relieving urine protein excretion (UPE) [mean difference (MD) = -0.35, 95% confidence interval (CI): -0.41 to -0.30, *P* < 0.00001] compared with the full-dose corticosteroids group. Regarding serum creatinine (SCr), estimated glomerular filtration rate (eGFR), complete remission rate, and overall response rate, there was no difference between the groups (*p* > 0.05). Regarding safety, low-dose corticosteroids combined with leflunomide significantly reduced the risk of serious adverse events [odds ratio (OR): 0.11, 95% CI: 0.01 to 0.91, *P* = 0.04]. Besides, no significant differences were observed between the two groups in the incidence of respiratory infection, abnormal liver function, diarrhea, herpes zoster, alopecia, pruritus, insomnia, pneumonia, diabetes, and urinary tract infection (*P* > 0.05).

**Conclusions:**

Low-dose corticosteroids combined with leflunomide are a safe and effective treatment for progressive IgA nephropathy.

**Trial registration:**

The PROSPERO registration number is CRD42022361883.

**Supplementary Information:**

The online version contains supplementary material available at 10.1186/s12894-024-01438-3.

## Introduction

Immunoglobulin A (IgA) nephropathy (IgAN) is a glomerular disease characterised by IgA or IgA-dominated immune complexes deposited in the glomerular mesangium [1]. Since Berger and Hinglais described it in 1968 [2], IgAN has become the most common primary glomerulonephritis [3]. The incidence of IgAN varies markedly in different regions, with the highest incidence in Asian countries [4, 5]. Although IgAN is considered a benign disease, studies have shown that approximately 30-45% of IgAN patients progress to end-stage renal disease (ESRD) within 20 years of onset with the need for renal replacement therapy [6, 7]. The current treatment for IgAN remains in the exploratory stage of development. Angiotensin-converting enzyme inhibitors (ACEIs) or angiotensin receptor blockers (ARBs) were recommended as first-line treatment for IgAN according to Kidney Disease Improving Global Outcomes (KDIGO) guidelines. In contrast, high-dose systemic corticosteroid therapy for six months is recommended for patients with proteinuria > 1 g/day and eGFR > 50 mL/min/1.73 m^2^ [8]. However, patients usually suffer many side effects from applying long-term and high-dose corticosteroids [9]. Therefore, control of corticosteroid dose is essential for the treatment of IgAN.

In recent years, immunosuppressants have gained attention as an adjuvant treatment option for IgAN [10, 11]. Leflunomide is a synthetic isoxazole derivative immunosuppressant that suppresses lymphocyte and B-cell proliferation by inhibiting pyrimidine biosynthesis [12, 13]. The effectiveness of leflunomide in treating rheumatoid arthritis, kidney disease, and organ transplant rejection has now been established [14]. Previous studies reported that leflunomide could significantly improve proteinuria and renal function deterioration in IgAN patients [15, 16]. However, few meta-analyses have been performed on the feasibility of leflunomide combined with corticosteroids for treating IgAN.

Therefore, we performed a meta-analysis to evaluate the efficacy and safety of low-dose corticosteroids combined with leflunomide for progressive IgAN. As far as we know, this meta-analysis reported the treatment effects of this combination for the first time.

## Methods

### Protocol

This meta-analysis has been registered on PROSPERO with registration number CRD42022361883. As the research method, our study adopted preferred reporting items for systematic reviews and meta-analyses (PRISMA) checklist [17].

### Search strategy

The article search used the PubMed, Embase, and Cochrane Library databases with the keywords “IgA nephropathy”, “leflunomide”, and “corticosteroids”. Our search strategy is based on PICOS (populations, interventions, comparators, outcomes, and study designs), as detailed in Table 1. We only considered published research (until Sept 2022). The entire search process was completed independently by three authors.

**Table 1:** Search strategy according to populations, interventions, comparators, outcomes, and study designs (PICOS)

	Population	Intervention	Comparator	Outcomes	Study Design
Inclusion Criteria	Patient was diagnosed as IgAN by renal biopsy	low-dose corticosteroid plus leflunomide	full-dose corticosteroid	eGFR (ml/min/1.73m^2^). Serum creatinine. Urine protein excretion. Complete remission. Overall remission. Complications.	Clinical research
Exclusion Criteria	Patients with rapidly progressive IgA nephropathy. Secondary IgA nephropathy due to the patient’s systemic illness. Patients with severe infections. Patients with Patients were on CS or other immunosuppressive therapy within 6 months before enrollment. Patients with malignancy, HIV infection, or acute central nervous system diseases.	Not performed	Not performed	Blood pressure. Hematuria. End-stage renal disease (ESRD). Acute kidney injury. Edema.	Letters, comments, reviews, qualitative studies

*IgAN* IgA nephropathy, *eGFR* Estimated glomerular filtration rate

### Inclusion criteria and trial selection

**Fig. 1:**
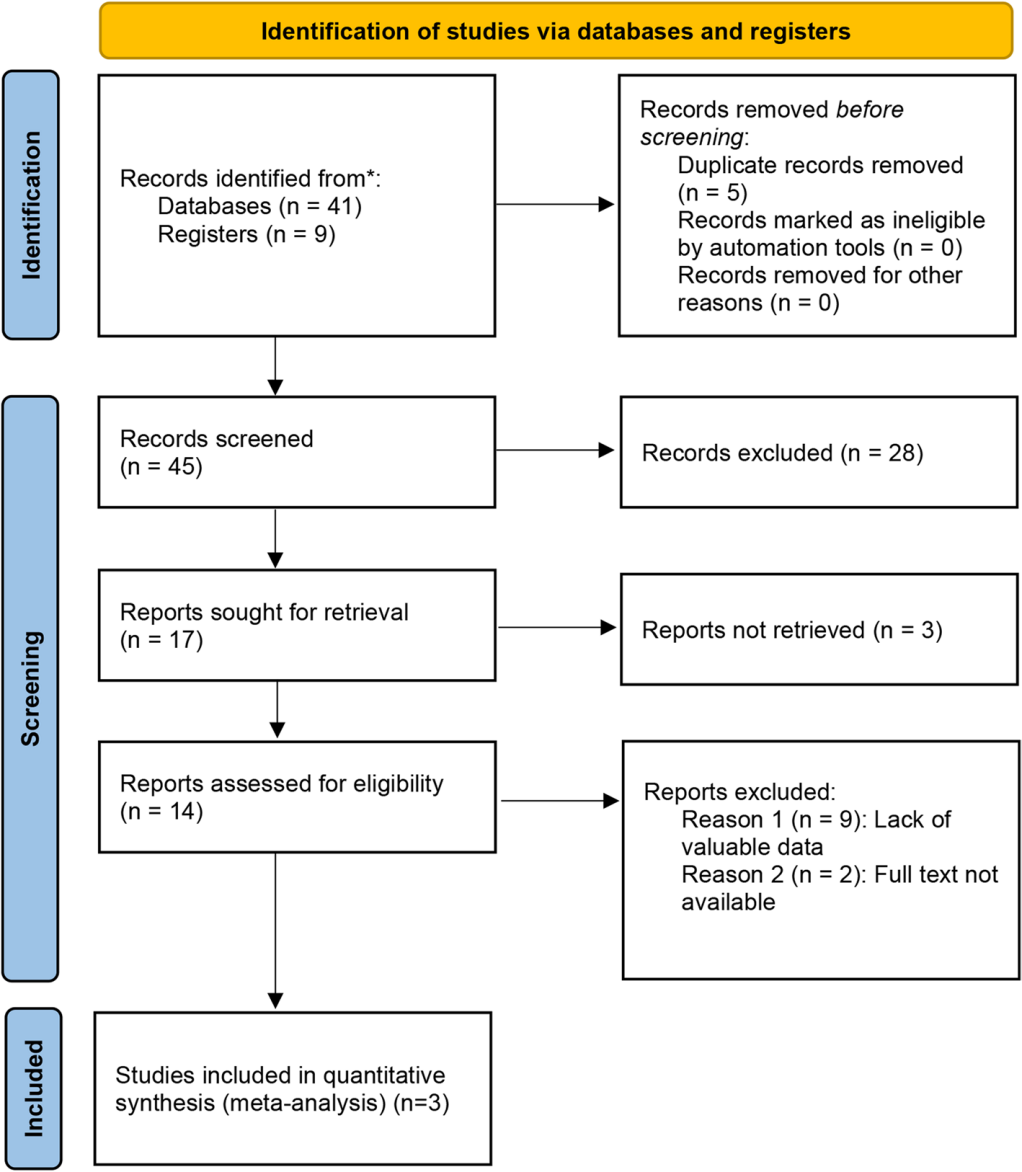
PRISMA of the study selection process

To be qualified for inclusion in our meta-analysis, included studies were required to meet the following criteria: (I) the study examined the effect of low-dose corticosteroids combined with leflunomide on IgAN; (II) the study contained sufficient valuable data, including the number of patients enrolled and the results of each observed indicator; (III) full text is available; (IV) the type of study was a clinical trial. We analysed the most recent study of identical reports published in a different journal. The process of inclusion and exclusion is outlined in Fig. 1, a PRISMA flowchart. The PRISMA 2020 checklist is supplied in the Supplemental material “PRISMA Checklist.”

### Quality assessment

The randomized controlled trials (RCTs) were assessed according to guidelines published in the Cochrane Handbook for Systematic Reviews of Interventions v.5.1.0 [18]. For the retrospective cohort study, the methodological index for nonrandomised studies (MINORS) score was used for evaluation [19]. Three independent investigators conducted a quality assessment of the included studies, and disagreements were resolved by discussion. Each study was graded for quality as (+) low risk of bias, (?) unclear risk of bias, and (-) high risk of bias. Quality assessment of non-randomized controlled trials was undertaken by MINORS score. The quality of evidence was classified as 0–12 for low quality, 13–18 for moderate quality, and 19–24 for high quality.

### Data extraction

The following information extracted from each included study was: (I) the first author’s name; (II) the study type; (III) the sample size (IV) the administration strategy; (V) the timing and dosage of medication; (VI) the other medications; (VII) the evaluation indicators, including urine protein excretion, serum creatinine, eGFR, serious adverse events, respiratory infection, abnormal liver function, diarrhea, herpes zoster, alopecia, pruritus, insomnia, pneumonia, diabetes, and urinary tract infection.

### Statistical and meta-analysis

All analyses used the statistical software Review Manager (RevMan, version 5.3.0, Cochrane Collaboration) [20]. For continuous data, we employed the mean difference (MD) with their corresponding 95% confidence intervals (95% CIs) for evaluation. Dichotomous data were evaluated by odds ratio (OR) with 95% CIs [21]. If the *P* value was more significant than 0.05, the meta-analysis estimate was pooled using a fixed-effects model with between-study heterogeneity quantified using the I^2^ statistic. A random effects model was used otherwise.

## Results

### Study selection and characteristics of the trials

According to the inclusion criteria, we obtained 50 articles from the databases and registers. 4 articles were considered duplicates and excluded. After browsing through the titles and abstracts, 28 articles were eliminated. 9 articles were excluded because of a lack of valuable data. In addition, two articles were excluded because the full text was unavailable. Ultimately, three studies were included in our meta-analysis [22–24], with two RCTs [22, 23]. Table 2 summarises the patient characteristics of the three studies.

**Table 2:** Study and patient characteristics

Study	Country	Design	Therapy in experimental group	Therapy in control group	Simple size		Usage of medication (mg/day)	Time of therapy (months)	Other medications	Inclusion population
					Trial	Control				
Min Lulin et al. (2017) [22]	China	RCT	low-dose prednisone plus leflunomide	full-dose prednisone	40	45	LEF group: LEF, 40 (3d) -- 20 (20 m); CS, 0.8 mg/kg/day (4-6w, max 40 mg/d). CS group: CS, 1 mg/kg/day (8-12w, max 60 mg/d).	12	ACEI/ARB	All patients had biopsy-proven primary IgAN with renal biopsy samples examined independently by two pathologists. Patients aged 18–65 years were included if they had proteinuria ≥ 1.0 g/24 h and an estimated glomerular filtration rate (eGFR) ≥ 30 ml/ min/1.73m^2^ (calculated by CKD-EPI equation).
Li Yebei et al. (2021) [24]	China	retrospective cohort study	low-dose corticosteroid plus leflunomide	full-dose corticosteroid	65	84	LEF group: LEF, 50 (3d) -- 20 (6 m); CS, 0.4 ~ 0.6 mg/kg/day (2 m) -- 0.32 ~ 0.48 mg/kg/day (4 m). CS group: CS, 0.8 ~ 1 mg/kg/day (2 m) -- 0.64 ~ 0.8 mg/kg/day (4 m).	18	ARB	Patients met the following criteria: (1) IgAN diagnosed by renal biopsy; (2) an age range of 16–65 years; (3) 24-h urinary total protein (24 h UTP) level > 0.75 g, (4) estimated glomerular filtration rate (eGFR) ≥ 50 ml/min per 1.73 m^2^, and (5) a follow-up time was up to 18 months.
Ni Zhaohui et al. (2021) [23]	China	RCT	low-dose prednisone plus leflunomide	conventionally dose prednisone	59	49	LEF group: LEF, 40 (3d) − 20 (12 m); CS, 0.5 ~ 0.8 mg/kg/day (8-12w, max 40 mg/d). CS group: CS, 1 mg/kg/day (8-12w, max 60 mg/d).	12	ACEI/ARB	Patients were aged 18–65 with biopsy-confirmed primary IgAN in recent 3 months, and with any one of the following indications for progression in IgAN: 24-h UPE > 1.0 g/day; eGFR < 60 mL/min per 1.73 m^2^ (calculated by CKD-EPI equation); and renal histological lesions defined as Lee’s IV, or glomerulus and/or segmental sclerosis ≥ 40%.

*RCT* Randomized controlled trials, *LEF* Leflunomide, *CS* Corticosteroid, *ACEI* Angiotensin-converting enzyme inhibitor, *ARB* Angiotensin receptor blocker

### Risk of bias in the studies

Two included studies were RCTs, and one was a retrospective cohort study. Both RCTs were randomised, controlled, prospective, open-label, controlled trials. Each RCT study described the randomisation process in detail, while the concealment procedures were not sufficiently described. Based on the MINORS, the included retrospective cohort study scores were 19 and considered high-quality. The results regarding the quality assessment are presented in Table 3.

**Table 3:** Quality assessment of individual study

Study	Random sequence generation (selection bias)	Allocation concealment (selection bias)	Blinding of outcome assessment (detection bias)	Blinding of participants and personnel (performance bias)	Incomplete outcome data (attrition bias)	Selective reporting (reporting bias)	Other bias	Statistical analysis	MINORS score
Min Lulin et al. (2017) [22]	**+**	**?**	**+**	**-**	**+**	**?**	**+**	T-test or ANCOVA	NA
Li Yebei et al. (2021) [24]	**-**	**-**	**?**	**-**	**?**	**?**	**+**	T-test or Pearson chi-square test	19 points
Ni Zhaohui et al. (2021) [23]	**+**	**?**	**+**	**-**	**+**	**?**	**+**	T-test or ANCOVA	NA

*ANCOVA* Analysis of covariance, *NA* Not applicable; 0–12 points, low quality; 13–18 points, medium quality; 19–24 points, high quality

### Efficacy

We determined the effectiveness of low-dose corticosteroids combined with leflunomide by comparing the impact of low-dose corticosteroids combined with leflunomide (Low CS + LEF) versus full-dose corticosteroids (Full CS) therapy in patients with IgAN. Complete remission refers to 24-h urine protein excretion < 0.4 g/d with a stable serum creatinine level (No more than 30% of baseline level).

### Urine protein excretion (UPE)

**Fig. 2:**
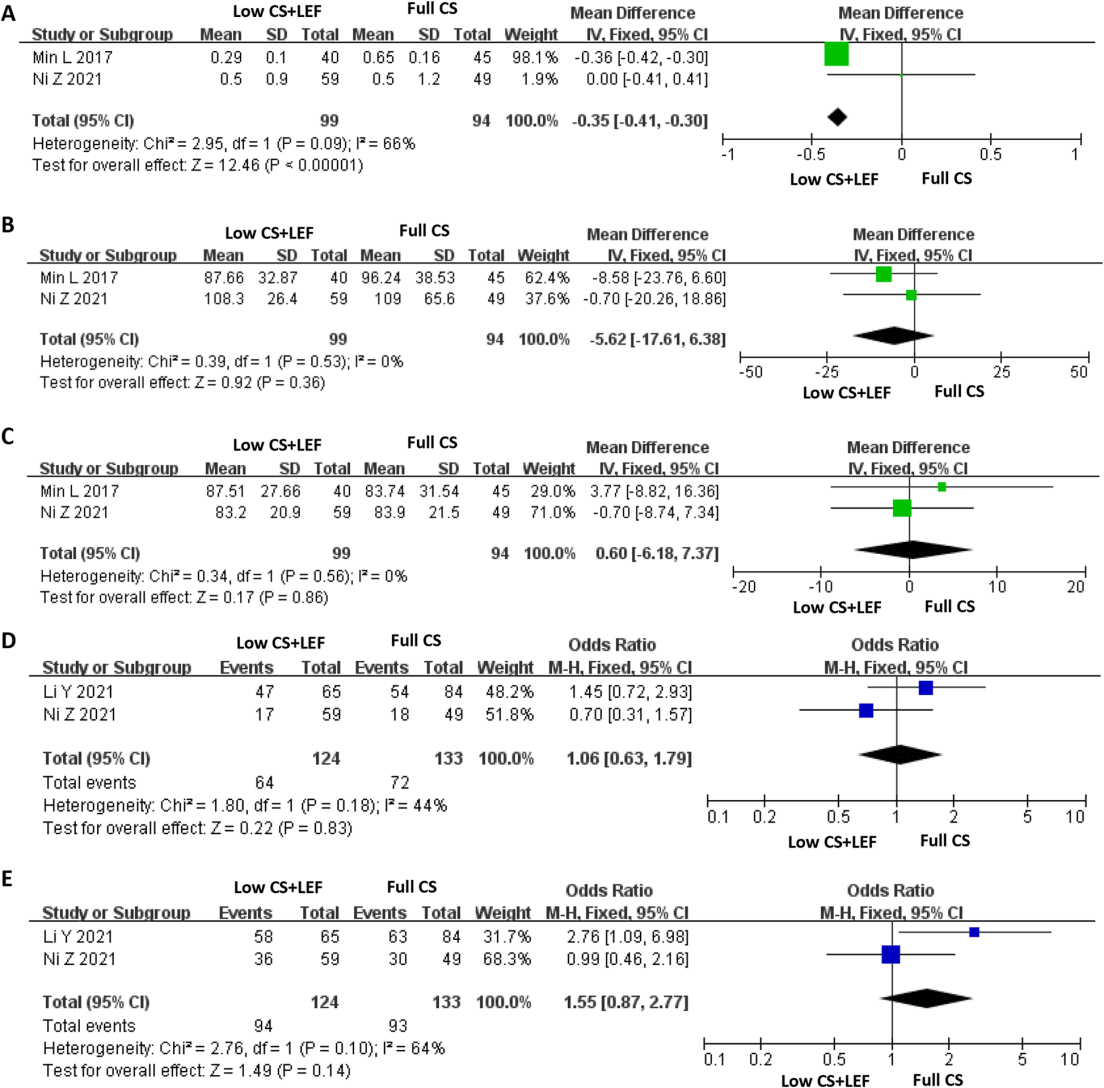
Forest plots showing changes in: **A** Urine protein excretion (UPE); **B** Serum creatinine (SCr); **C** Estimated glomerular filtration rate (eGFR); **D** Complete remission; **E** Overall response

We found two RCTs encompassing 193 patients, including 12-month follow-up data (99 in the Low CS + LEF group, 94 in the Full CS group) that evaluated the UPE. The pooled data displayed an MD of − 0.35 and a 95% CI of − 0.41 to -0.30 (*P* < 0.00001) from a fixed-effects model (Fig. 2A). Results demonstrated that the UPE was significantly decreased in the Low CS + LEF group compared with the Full CS group.

### Serum creatinine (SCr)

Two RCTs (193 patients; 99 in the Low CS + LEF group, 94 in the Full CS group) included data after 12 months of follow-up on the SCr. Fixed-effects models revealed no differences in Scr levels observed between the two groups (MD = − 5.62, 95% CI: −17.61 to 6.38, *P* = 0.36) (Fig. 2B).

### Estimated glomerular filtration rate (eGFR)

A total of two RCTs recorded the eGFR in 193 patients. We conducted a fixed-effects model for the analysis (Fig. 2C). The heterogeneity test showed *P* = 0.56 and I^2^ = 0%. The 12-month follow-up information of the included patients. The findings demonstrated that the eGFR did not differ significantly between the Low CS + LEF and Full CS groups (MD = 0.60, 95% CI: −6.18 to 7.37, *P* = 0.86).

### Complete remission

The forest plot yielded an OR of 1.06 with a 95% CI of 0.63 to 1.79 (*P* = 0.83), which suggested that the Low CS + LEF group and Full CS group were similar regarding complete remission (Fig. 2D). The 18-month follow-up information of the included patients.

### Overall response

The forest plot yielded an OR of 1.55 with a 95% CI of 0.87 to 2.77 (*P* = 0.14), which concluded that there was no difference between the two groups in overall response (Fig. 2E). The 18-month follow-up information of the included patients.

### Safety

#### Serious adverse events

**Fig. 3:**
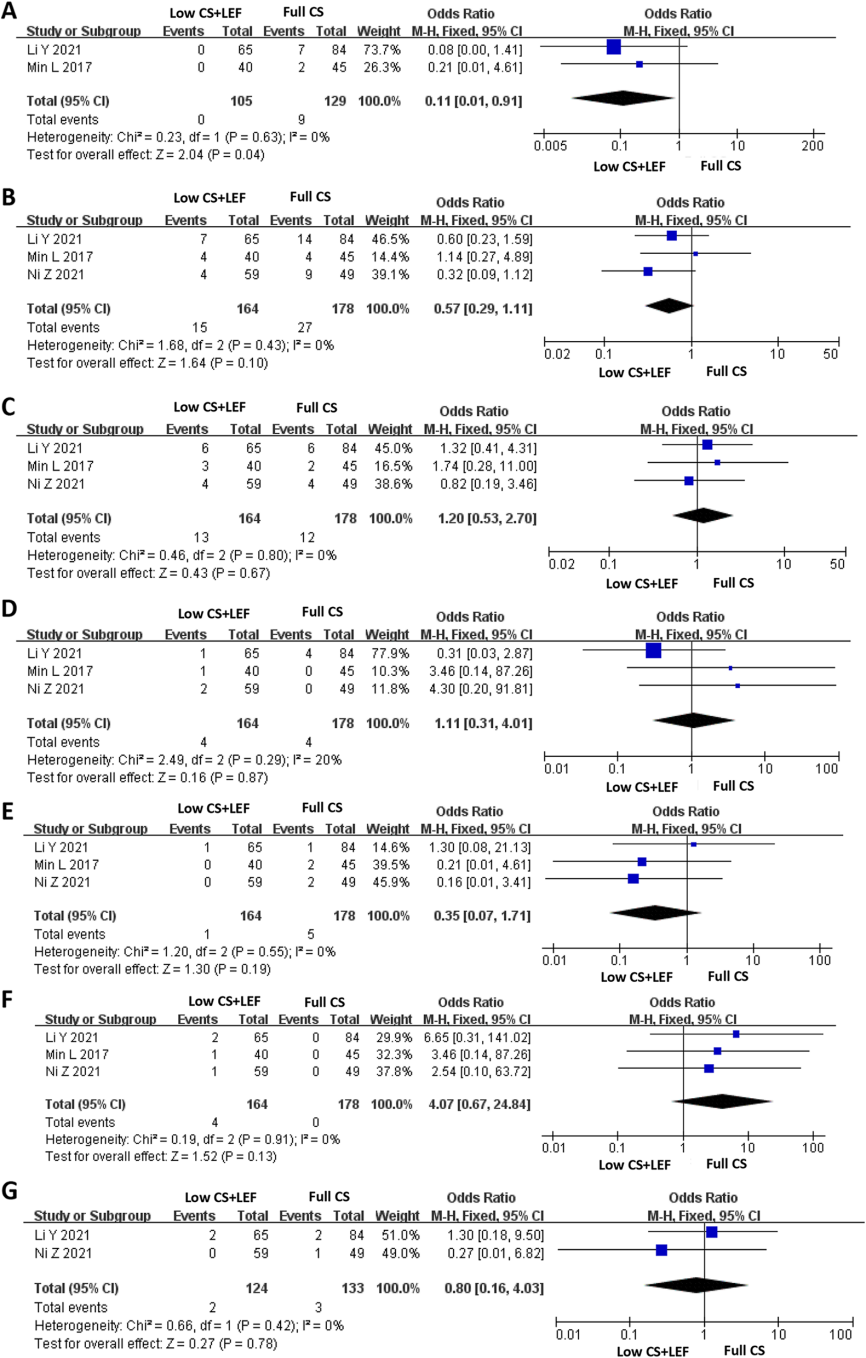
Forest plots showing changes in: **A** Serious adverse events; **B** Respiratory infection; **C** Abnormal liver function; **D** Diarrhea; **E**, Herpes zoster; **F** Alopecia; **G** Urinary tract infection

Two studies, including 342 patients with at least 12 months of follow-up (164 in the Low CS + LEF group and 178 in the Full CS group), reported severe adverse events. The results from a fixed-effects model demonstrated that the Low CS + LEF group had an advantage in improving the incidence of serious adverse events (OR = 0.11, 95% CI: 0.01 to 0.91, *P* = 0.0.04) (Fig. 3A).

#### Respiratory infection

Three studies analysed the incidence of respiratory infection in 342 patients. The 18-month follow-up information of the included patients. We conducted a fixed-effects model for the analysis (Fig. 3B). The heterogeneity test showed *P* = 0.43 and I^2^ = 0%. The findings demonstrated no significant difference between the two groups in respiratory infection (OR = 0.57, 95% CI: 0.29 to 1.11, *P* = 0.10) (Fig. 3B).

#### Abnormal liver function

Three studies (342 patients; 164 in the Low CS + LEF group, 178 in the Full CS group) recorded the risk of abnormal liver function. The 18-month follow-up information of the included patients. Fixed-effects models revealed no differences in liver function were observed between the two groups (OR = 1.20, 95% CI: 0.53 to 2.70, *P* = 0.67) (Fig. 3C).

#### Diarrhea

Three studies, including 342 patients, examined the risk of diarrhea. The 18-month follow-up information of the included patients. A fixed-effects model was utilised to analyse the data. Based on the analysis results, we found no significant differences between the Low CS + LEF group and the Full CS group for diarrhea (OR = 1.11, 95% CI: 0.31–4.01, *P* = 0.87, Fig. 3D).

#### Herpes zoster

Three studies, including 342 patients with at least 12 months of follow-up data, reported the incidence of herpes zoster. We conducted a fixed-effects model for the analysis (Fig. 3E). Based on the analysis results, we suggested no significant difference between the two groups in herpes zoster (OR = 0.35, 95% CI: 0.07–1.71, *P* = 0.19).

#### Alopecia

Three studies analysed alopecia in 342 patients with at least 12 months of follow-up data. We conducted a fixed-effects model for the analysis (Fig. 3F). The findings demonstrated no significant difference between the two groups in alopecia (OR = 4.07, 95% CI: 0.67 to 24.84, *P* = 0.13) (Fig. 3F).

#### Urinary tract infection

The forest plot identified an OR of 0.80 with a 95% CI of 0.16 to 4.03 (*P* = 0.78), which suggested that the Low CS + LEF group and Full CS group were similar regarding urinary tract infection (Fig. 3G). The 18-month follow-up information of the included patients.

#### Pruritus and insomnia

Two RCTs involving 193 patients with at least 12 months of follow-up data (99 in the Low CS + LEF group, 94 in the Full CS group) had data on pruritus and insomnia. The pooled results from the fixed-effects model indicated that the Low CS + LEF group and Full CS group were similar in terms of pruritus (OR = 3.91, 95% CI: 0.42 to 35.98, *P* = 0.23) and insomnia (OR = 0.24, 95% CI: 0.03 to 2.19, *P* = 0.21) (Supplementary Figures 1 and 2).

#### Pneumonia and diabetes

Two studies, including 234 patients (105 in the Low CS + LEF group and 129 in the Full CS group), contained data on pneumonia and diabetes. The 12-month follow-up information of the included patients. With a fixed effects model, the OR for pneumonia was 0.46 (95% CI, 0.10–2.03, *P* = 0.30), and the OR for diabetes was 0.40 (95% CI, 0.09–1.73, *P* = 0.22). Based on the above results, we found no significant differences in pneumonia and diabetes between the two groups (Supplementary Figures 3 and 4).

## Discussion

At present, the aetiology and pathogenesis of IgAN remain controversial. Because mechanisms leading to disease are likely multifactorial, no standard treatment for IgAN patients currently exists. Its corresponding treatment mainly relies on ACEIs and CS, which have inconsistent effects [25]. Moreover, the adverse effects caused by the long-term application of corticosteroids, such as infection, abnormal glucose and lipid metabolism, and femoral head necrosis [9], limit the application of corticosteroids. Therefore, a refinement of treatment and alternative treatment protocols is much needed.

A growing body of evidence supports the contribution of immunosuppressants in the treatment of IgAN [26, 27]. Leflunomide can be rapidly converted in vivo to active metabolites, inhibiting the production and action of inflammatory mediators and cytokines inextricably linked to kidney disease [16]. These may be the mechanism of leflunomide in treating IgAN. Besides that, combination protocols that include leflunomide have shown significant advantages in efficacy and safety. Lv et al. [28] reported the beneficial effects of combined treatment with leflunomide and corticosteroids for IgAN. In an RCT, Cheng et al. [15] demonstrated that valsartan combined with clopidogrel and leflunomide could protect renal function with minimal adverse effects.

Our meta-analysis focused on the efficacy and safety of low-dose corticosteroids combined with leflunomide for progressive IgA nephropathy. Many studies have shown that urinary protein can cause damage to renal tubular epithelial cells. Thus, proteinuria is considered a risk factor for IgAN progression. Xie et al. [29] reported that relief of proteinuria is essential for long-term renal function protection in IgAN patients. Our results showed that low-dose corticosteroids combined with leflunomide were superior to full-dose corticosteroids in improving urine protein excretion. The SCr and eGFR are also associated with the prognosis of IgAN patients. The present study concluded that there appeared to be no difference between these two treatment options concerning SCr, eGFR, complete remission rate, and overall response rate. In addition, the present study found that low-dose corticosteroids plus leflunomide significantly reduced the incidence of serious adverse events compared to full-dose corticosteroids, suggesting that this combination protocol was relatively safe for treating progressive IgAN.

In contrast to previous studies [28], our study focuses more on the safety and efficacy of low-dose corticosteroids combined with leflunomide in treating progressive IgA nephropathy. In addition, our study also found that the low-dose corticosteroids combined with leflunomide significantly reduced UPE and the incidence of serious adverse events, which was not mentioned in the previous study. Lower doses of corticosteroids minimised adverse effects, and the combination with leflunomide did not affect therapeutic efficacy. This program may be a preferable alternative for IgAN patients with full-dose corticosteroid-associated contraindications. In a mouse model of IgAN, leflunomide and corticosteroids reduced deposition of the glomerular mesangial immune complex, with leflunomide exhibiting a more pronounced effect [30].

Our study had certain limitations. First, a relatively small number of patients was included in the study. We will continue to follow the latest RCTs, allowing us to comprehensively address this limitation in the future. Second, the included studies had qualitative weaknesses, mainly in study design, allocation concealment, and blinding. These limitations may lead to the risk of selection bias and information bias. So, our results should be interpreted with caution. Third, the other limitation of our study is that most patients included were from Asian populations. Therefore, there may be ethnic differences in our findings, and further studies are still needed to evaluate the effect of this combination therapy in a global population. Fourth, the follow-up period was brief and subsequent analyses with long-term follow-up were necessary. A more significant number of high-quality RCTs would still be needed to validate our conclusions.

## Conclusions

This meta-analysis suggested that low-dose corticosteroids combined with leflunomide for progressive IgAN provide similar results to full-dose corticosteroids and have advantages in relieving urinary protein and reducing SAEs. This protocol promises to be a new option for treating progressive IgAN.

### Supplementary Information

**Supplementary Material 1.**

**Supplementary Material 2.**

## Data Availability

The datasets generated during and/or analyzed during the current study are available from the corresponding author on reasonable request.

## Abbreviations

UPE: Urine protein excretion
MD: Mean difference
CI: Confidence interval
Scr: Serum creatinine
OR: Odds ratio
IgAN: Immunoglobulin A nephropathy
ESRD: End-stage renal disease
ACEIs: Angiotensin-converting enzyme inhibitors
ARBs: Angiotensin receptor blockers
KDIGO: Kidney Disease Improving Global Outcomes
PRISMA: Preferred reporting items for systematic reviews and meta-analyses
MINORS: Methodological index for nonrandomized studies
RCTs: Randomized controlled trials
CS: Corticosteroids
LEF: Leflunomide
