# Supplementary material for: Efficacy and safety of low-dose corticosteroids combined with leflunomide for progressive IgA nephropathy: a systematic review and meta-analysis
Source: BMC Urol. 2024 Mar 11;24:56. doi: 10.1186/s12894-024-01438-3 (PMC10926645; doi:10.1186/s12894-024-01438-3)
Supplement: Supplementary file 2 — Supplementary Material 2. [file 12894_2024_1438_MOESM2_ESM.docx]

**Title**: Efficacy and Safety of low-dose corticosteroids combined with leflunomide for progressive IgA nephropathy: A Systematic Review and Meta-Analysis


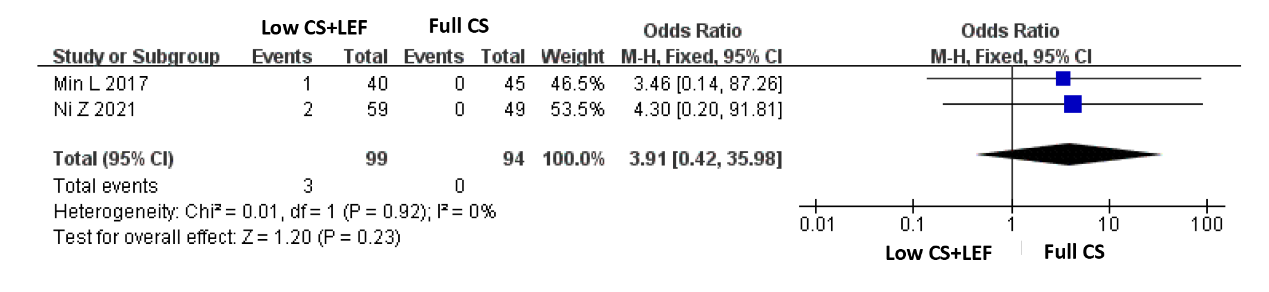


**Supplementary Figure 1.** Forest plots showing changes in pruritus.


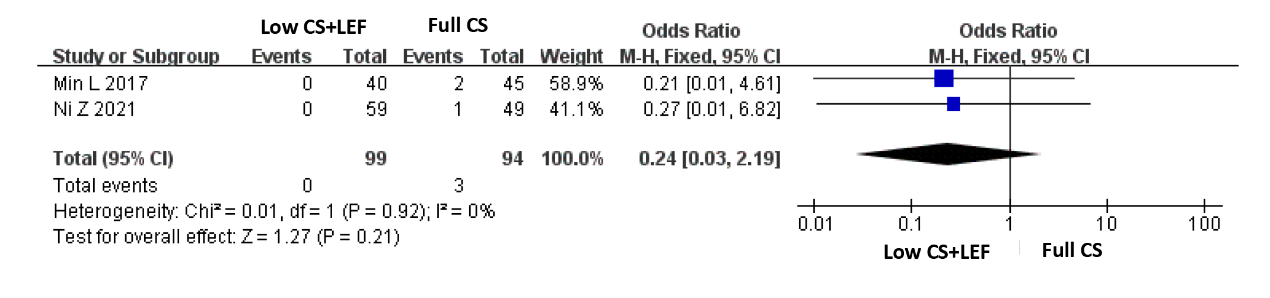


**Supplementary Figure 2.** Forest plots showing changes in insomnia.


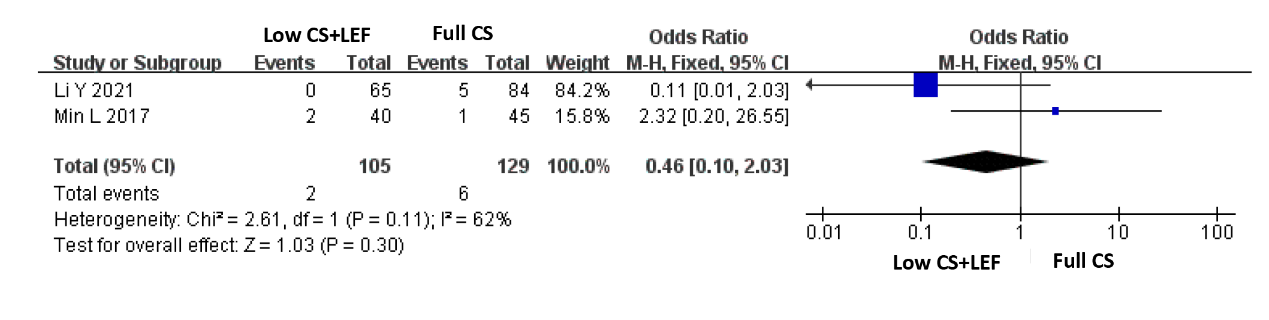


**Supplementary Figure 3.** Forest plots showing changes in pneumonia.


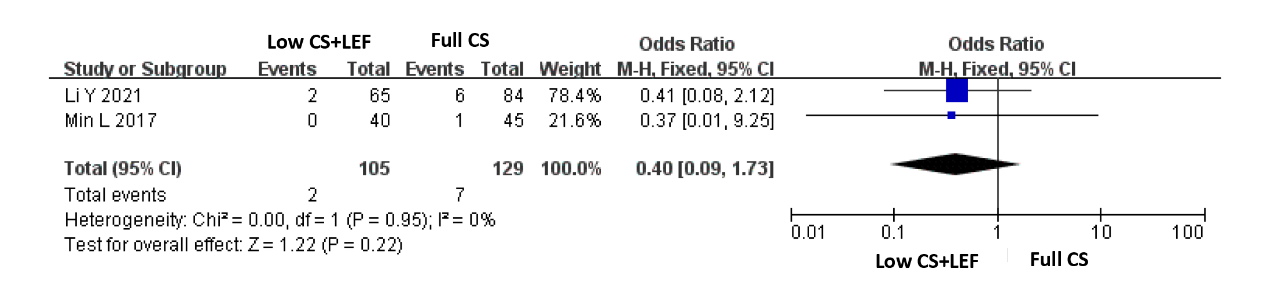


**Supplementary Figure 4.** Forest plots showing changes in diabetes.
